# The impact of fine motor activities like playing musical instruments on the thickness and strength of the flexor digitorum muscle

**DOI:** 10.1186/s12995-024-00430-9

**Published:** 2024-08-14

**Authors:** Christos I. Ioannou, Franziska L. Hodde-Chriske, Marios N. Avraamides, Eckart Altenmüller

**Affiliations:** 1 CYENS Centre of Excellence, 01 Dimarchou Lellou Demitriade Square, Nicosia, 1016 Cyprus; 2 Institute of Music Physiology and Musicians’ Medicine, Hanover University of Music, Drama and Media, Hanover, Germany; 3 Hanover Medical School, Hanover, Germany; 4 Department of Psychology, University of Cyprus, Nicosia, Cyprus

**Keywords:** Occupational activities, Ultrasound, Muscle adaptation, Side-by-side asymmetry

## Abstract

**Background:**

This study aimed to explore the impact of occupational activities involving extensive finger movement on the muscular characteristics of the forearms. In particular, the flexor digitorum (FD) muscular thickness and strength asymmetry between right and left hand were compared between musicians and non-musicians.

**Methods:**

Ultrasonography was employed to measure the thickness of the FD in each hand, while a validated custom-made device was used to assess the strength of the flexor and extensor digitorum (ED). Initially, muscle differences were estimated by computing the asymmetry index between dominant and non-dominant hands. To assess potential occupational disparities, comparisons of the asymmetry index were conducted between 25 right-handed instrumental musicians and 25 right-handed non-musicians.

**Results:**

Musicians exhibited lower asymmetry between dominant and non-dominant hands in both FD thickness and ED strength when compared to non-musicians. This effect was particularly pronounced in musicians playing instruments that extensively use the left-hand fingers (e.g., violinists).

**Conclusions:**

Occupational activities, such as playing a musical instrument, can alter forearm muscle mass and strength distribution between dominant and non-dominant hands. This underscores the importance of considering occupational parameters in clinical or experimental interventions and musculoskeletal assessments.

**Supplementary Information:**

The online version contains supplementary material available at 10.1186/s12995-024-00430-9.

## Background

Muscular imbalances between dominant and non-dominant sides have been reported in various professions such as athletes [1–3] and dancers [4, 5]. However, it remains unclear whether such imbalances can be generalized to professions requiring extensive finger fine-motor control during un-ergonomic positions. For instance, violinists, who use extensively the fingers of their left hands could exhibit different finger muscle asymmetry between dominant and non-dominant hands than trumpet players, who use extensively only the fingers of their right hand. Although there may not be an immediate pathological concern, in clinical practice, it is often observed that double-bass players tend to develop very strong finger flexor muscles in the left forearm due to the physical demands of pressing down the strings. However, several studies have revealed that long-term muscular asymmetries in musicians serve as a potential risk factor for the development of musculoskeletal injuries [6]. Consequently, it is imperative to consider these side-by-side muscular imbalances in preventive and clinical procedures, as well as in research studies where various ergonomically challenging occupations or musicians playing different instruments are grouped together [7].

Some of the muscles often affected by overuse or various medical conditions in musicians are the superficial and profound flexor digitorum (FD) muscles. These muscles are responsible for the flexion of the fingers (digits II to V). Specifically, the superficial part of the FD flexes the middle phalanges, whereas the profound part flexes the distal phalanges of the fingers [8, 9]. In musicians, the FD (including superficial and profound parts) is frequently affected by extensive finger overuse, which can lead to the development of fatigue and chronic pain as well as more critical neurological conditions such as focal hand dystonia (also known as musician’s dystonia in musicians) [10, 11].

Specific non-muscular side-by-side asymmetries among instrumental musicians have also been reported in different domains. For instance, Kopiez et al. [12] reported a right-hand skill superiority in left-handed pianists that was positively associated with practice time. Bangert et al. [13] found that the precentral gyrus, a brain region associated with hand and finger movements, was larger in the right hemisphere of musicians (e.g., violinists), which is responsible for controlling movements of the left side of the body. The authors argued that this structural brain difference might reflect an adaptation to the specific demands of the different musical instruments [14]. However, muscular asymmetry differences between dominant and non-dominant sides, such as muscle thickness and strength, have yet to be documented in instrumental musicians. To address this gap in the literature, the current study compared muscle asymmetries between dominant and non-dominant hands across musicians and non-musicians based on (a) the thickness and strength of the flexor digitorum (FD) (including both superficial and profound parts), and (b) the strength of the extensor digitorum as well (ED). The thickness of the FD was measured via ultrasonography, a validated and reliable method for assessing muscular thickness [15–19]. The strength of the FD and the ED was measured using a validated device developed in our clinic [17].

We hypothesize that a smaller asymmetry between dominant and non-dominant hands will be observed in musicians’ thickness and strength of the FD, as well as strength of the ED, as compared to non-musicians. This asymmetry will be further affected by the different characteristics (playing requirements) of the different instruments.

## Methods

### Participants

Twenty-five right-handed healthy musicians and 25 right-handed healthy non-musicians were invited to participate. All participants were volunteers from local universities, orchestras, and music schools. Exclusion criteria included all orthopedic conditions and neurological disorders that are known to affect the muscular thickness and/or the strength of the upper limbs, e.g., lateral epicondylitis, tendonitis, carpal tunnel syndrome, radial tunnel syndrome, hand dystonia, etc. Demographic characteristics about the sample are presented in Table 1. The study was approved by the ethics committee of the Hanover Medical School and all participants gave their written consent prior to participation.

**Table 1:** Participants’ characteristics

Characteristics	Musicians (*n* = 25)	Non-Musicians (*n* = 25)	Group differences (sig.)
Age: (years) Median (min, max)	26 (19, 61)	26 (19, 54)	*p* > .05
Handedness: Right/Left/Ambidextrous (%)	100 / 0 / 0	100 / 0 / 0	-
Handedness percentage: (%), Median (min, max)*	90 (50, 100)	100 (60, 100)	*p* > .05
Body Mass Index: (kg/m^2^), Median (min, max)	21.7 (17.2, 36.6)	22 (17.4, 27.8)	*p* > .05
Gender: Male / Female (%)	32 / 68	40 / 60	*p* > .05
Musical Genre: Classic / Jazz, Pop, Rock / Other (%)	84.6 / 7.7 / 7.7	-	-
Cumulative hours of practicing:		-	-
- *Primary instrument: Median (min*,* max)*	10,587 (2556, 67527)	-	-
- *Secondary instrument: Median (min*,* max)*	4015 (183, 40150)	-	-
Age started playing (years):			
- *Primary instrument: (M ± SD)*	7.3 ± 3.6	-	-
- *Secondary instrument: (M ± SD)*	11.3 ± 5.6	-	-
Years of experience:			
- *Primary instrument: (M ± SD)*	23.5 ± 11.1	-	-
- *Secondary instrument: (M ± SD)*	11.6 ± 4.2	-	-

No significant differences were found between musicians and non-musicians with respect to age (*U* = 308, *z* = − 0.088, *p* = .930), handedness percentage (*U* = 260, *z* = -1.099, *p* = .272), Body Mass Index (*U* = 272, *z* = − 0.786, *p* = .432) and gender (χ^2^ (1) = 0.347, *p* = .556). *Handedness percentage is defined as: left-handed <-40%, ambidextrous − 40 ≤ + 40, and right-handed > + 40. [20]

### Muscular thickness (Ultrasound)

The ultrasound measurement point on the forearm was located at 67% proximally from the styloid process of the radius towards the biceps tendon at the point of a bent elbow joint. Imaging was performed in both upper limbs while the participant was sitting upright with the shoulder joint flexed at about 45° anterior of the coronal plane and the forearm extended horizontally in a supinated position (for more details including photographic material see [7, 17]). Images (B-mode) were taken while the transducer (linear - SL1543, 3.0–13.0 MHz connected to a MyLabSix Ultrasound System, Esaote, Netherlands) was placed perpendicularly to the longitudinal axis of the forearm. The transducer was coated with water-based gel and pressure was reduced to the minimum to prevent compression of the muscle during imaging. Since both, superficial and profound FD muscles are involved in the flexion of the fingers [9, 16], and because the clear border between the two parts is sometimes hard to identify via ultrasonography (see supplementary material – Figure A) they were treated as one unit.

**Fig. 1:**
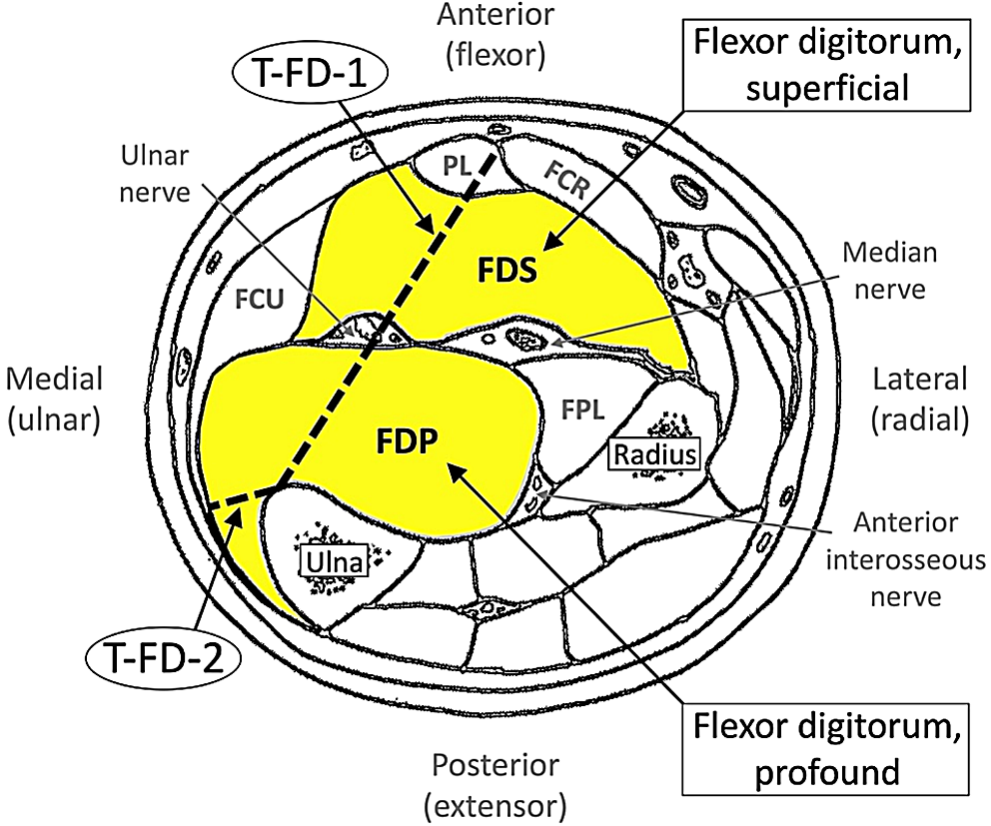
Ultrasound assessments of the muscular thickness of the flexor digitorum muscles (superficial and profound, assessed as one unit together). Abbreviations: FCR = flexor carpi radialis; FCU = flexor carpi ulnaris; FPL = flexor pollicis longus; PL = palmaris longus; T-FD-1 = first thickness assessment of the FD; T-FD-2 = second thickness assessment of the FD – 2nd measurement. The template of the current figure was received and modified with permission from Sklerov and Pullman [21] and the original source [22], Charles C Thomas Publisher, Ltd., Springfield, IL, USA

The overall thickness of the FD (T-FD) was computed by averaging two different thickness assessments aiming to cover different parts of the muscle. Both thickness assessments were captured from well-identifiable points: the first thickness assessment of the FD (T-FD-1) covered the distance between the ulnar side of the flexor carpi radialis muscle and the anterior margin of the ulna bone, whereas the second thickness assessment of the FD (T-FD-2) covered the distance between the ulnar side of the flexor carpi ulnaris muscle and the anterior margin of the ulna bone (Fig. 1). The decision to assess two different parts of the FD was based on the non-canonical shape of the muscle and due to the fact that the different finger fascicles (digits II to V) are located in different regions of the muscles (superficial and profound respectively), [9]. The specific method has been previously validated and yielded excellent reliability, ICC > 0.95. For more details including photographic material see [17].

### Muscular strength

The strength of the FD (S-FD) was computed by averaging the individual finger strength measurements (digits II to V) for the right and the left hand respectively. In addition, the average strength of the extensor digitorum (S-ED) was also computed from the individual finger strength measurements (digits II to V) of each hand. Assessments were performed while the palm, the wrist, and the lower arm, were attached to the custom-made device horizontally. Reliability of the current method was excellent, ICC > 0.91 [17].

### Procedure

Initially, specific landmarks in each forearm were identified according to Ioannou et al. [17] to specify the exact location of the transducer. Afterwards, the ultrasound measurements were taken, followed by the finger strength assessments which were divided into three different sessions. Between the strength sessions, participants filled in questionnaires, giving them time to relax. To minimize carryover effects within the strength assessment sessions, the order of the fingers’ strength measurements (II – V), the direction of the finger movement itself (flexion, extension), and the order of the hand (right, left) were randomized. Participants were instructed to perform each strength assessment at their maximum power. All ultrasound and strength assessments were performed three consecutive times and the average values were used during the analysis [17, 23]. The questionnaire, which was completed between the strength sessions included: (a) demographics, (b) medical and occupation-related information, and (c) the Edinburgh Handedness Inventory, [20] which assesses handedness.

### Statistical analysis

Bilateral asymmetry between hands was estimated using the Symmetry Index (SI): $\:\left(\frac{{X}_{D}-{X}_{ND}}{0.5*({X}_{D}+{X}_{ND})}\right)*100.$ Assuming that *X*_*D*_ > *X*_*ND*_, where *X*_*D*_ and *X*_*ND*_ indicate assessments of the dominant (D) and the non-dominant hand (ND) respectively, SI estimates as a percentage the level of asymmetry between the two hands. A distribution around 0% indicates no asymmetry whereas positive or negative percentages indicate larger thickness or strength to the right or to the left forearm muscles respectively [24].

The level of significance was set at *a* < 0.05 and Bonferroni corrections were applied in case of multiple comparisons. The assumptions of normality and homogeneity of variance were tested with Shapiro-Wilks and Levene’s tests respectively. For group comparisons, Independent Sample *t*-tests and One-way Analysis of variance (ANOVA) tests (including a Kruskal-Wallis test when necessary) were used. Finally, the Chi-squared test was used to determine differences between frequencies and the Pearson’s product moment correlation coefficient was used to test associations between variables. Effect sizes were estimated with Pearson’s correlation coefficient, *r*, and eta squared, $\:{\eta\:}_{\:}^{2}$.

One missing value was present in one of the two individual ultrasonographic thickness measurements (T-FD-1) for two musicians. As a result, the mean thickness of the FD (i.e., T-FD) and therefore the thickness asymmetry value between right and left hand could not be calculated. Consequently, data from the two participants were excluded for the specific variable.

## Results

**Fig. 2:**
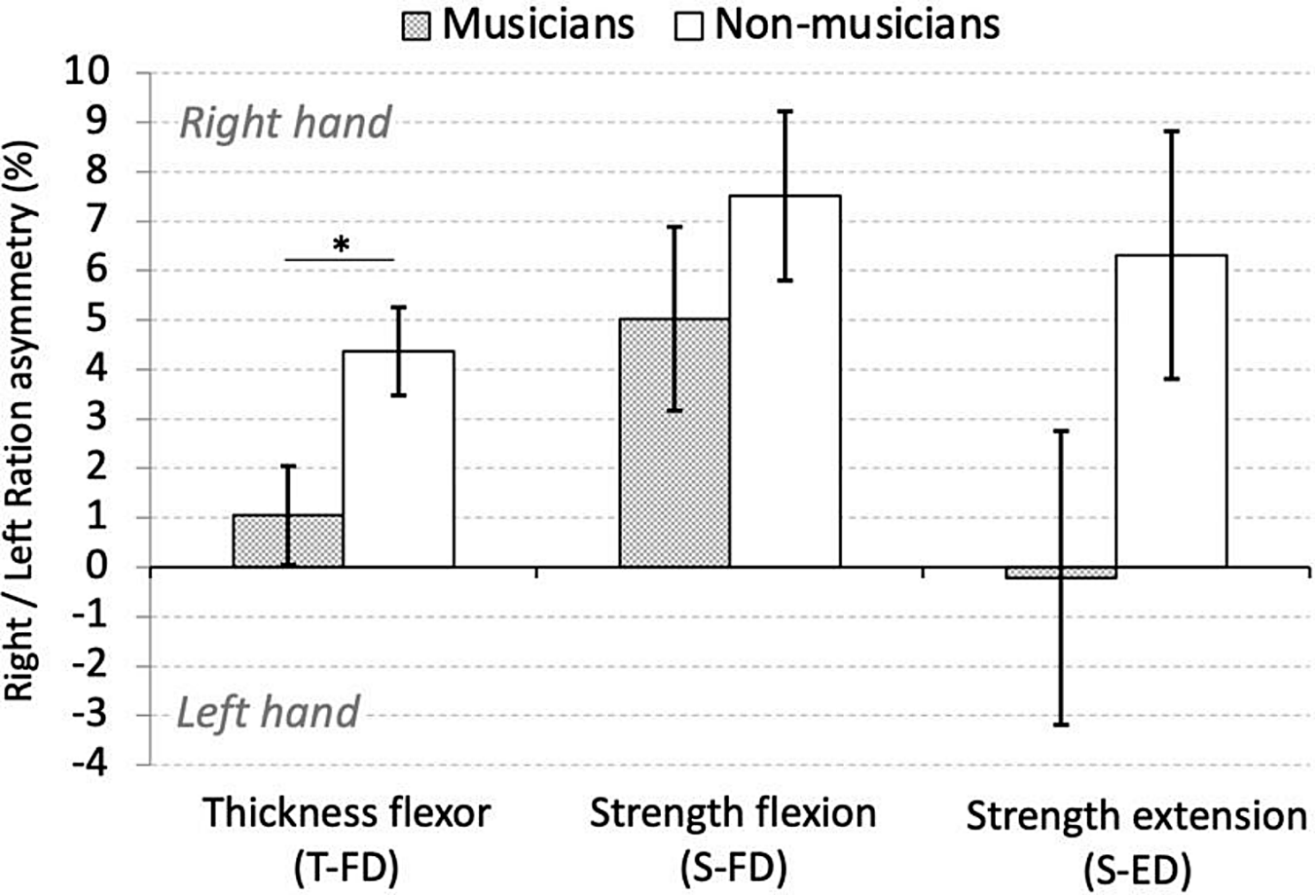
Ratio asymmetry between right and left hands between musicians and non-musicians. Zero indicates no asymmetry between right and left hand. Positive and negative values indicate larger asymmetry towards the right and left upper limb respectively. Asterisk indicates a significant group difference at the Bonferroni-adjusted *p* value (< 0.016). Error bars: ±1SE

Hand asymmetry differences between musicians and non-musicians for the T-FD, S-FD, and S-ED assessments are presented in Fig. 2. Results showed that compared to non-musicians, musicians had a more equal distribution between the right and left hand for the T-FD (*t*(46) = -2.488, *p* = .0165, *r* = .34) but not for the S-FD (*t*(48) = -0.983, *p* = .331, *r* = .14) or the S-ED (*t*(48) = -1.68, *p* = .09, *r* = .24). Significance was accepted at *p* < .05/3 = 0.016 (Bonferroni).

**Fig. 3:**
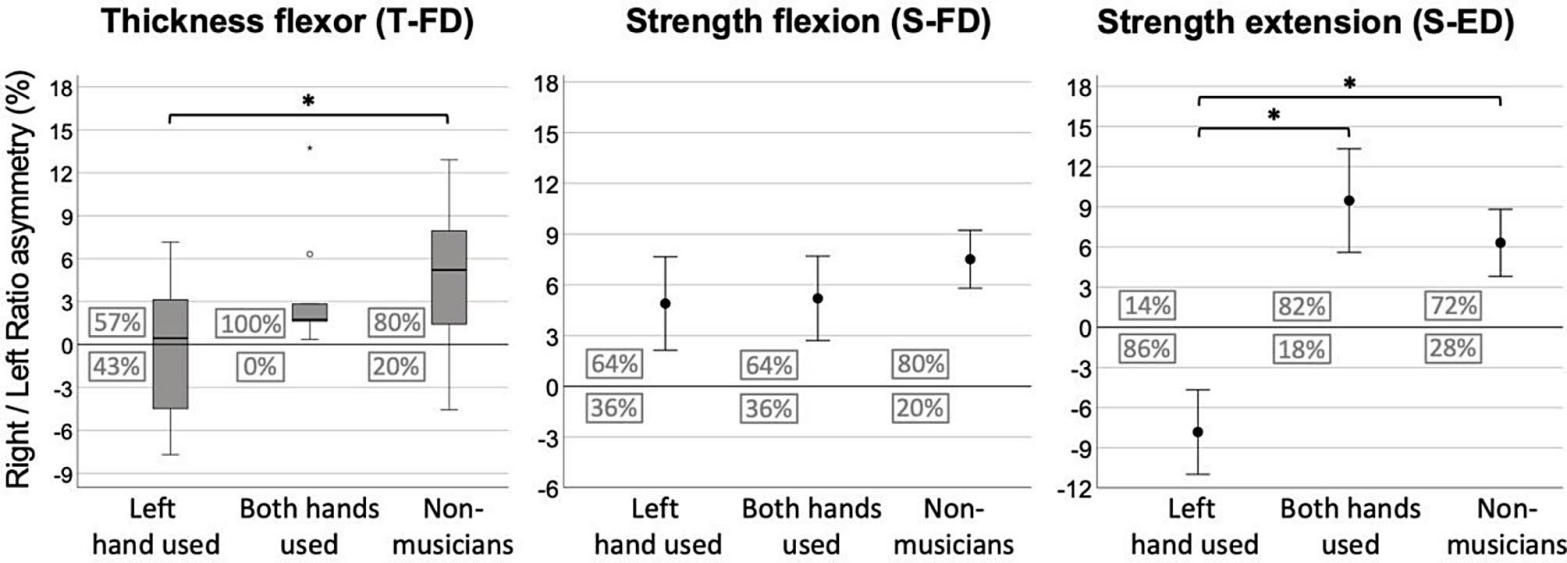
The group of musicians who primarily used the fingers of their left hand (*n* = 14) consisted of nine violinists, one violist and four cellists. The group of musicians who used the fingers of both hands equally (*n* = 11) consisted of five pianists, one flutist, one oboist, one bassoonist, one guitarist, one accordionist and one trombonist. No musicians were found who primarily used the fingers of their right hand (e.g., trumpet players). Two missing values were also reported in the T-FD assessments for the subgroup “both hands used”. Asterisk indicates post hoc significance tests (*p* < .05, Bonferroni). Error bars: ±1SE. Boxed values indicate the exact number of participants (expressed as a percentage) who showed higher asymmetry toward the right hand (above zero) or toward the left hand (below zero)

Finally, hand asymmetry differences were examined between (a) musicians who play their instrument using mainly the fingers of their left hand (e.g., violinists), (b) musicians who use both hands to the same degree (e.g., flutists), and (c) non-musicians. No musicians who mainly use the fingers or their right hand (e.g., trumpetists) were found. A Kruskal-Wallis test for the muscular thickness assessment (due to a violation of normality in the “both hands used” subgroup) and two ANOVAs for the flexion and extension strength assessments were conducted (Fig. 3). The T-FD (*H*(2) = 9.253, *p* = .01, $\:{\eta\:}_{}^{2}=0.20$) and the S-ED, (*F*(2,49) = 7.718, *p* = .001, $\:{\eta\:}_{}^{2}=0.25$) indicated significant group differences whereas the S-FD indicated no differences (*F*(2,49) = 0.477, *p* > .05, $\:{\eta\:}_{}^{2}=0.02$). Results were accepted as significant at *p* < .05/3 = 0.016 (Bonferroni). Post hoc significance tests are presented in Fig. 3.

Finally, the number of people (expressed as a percentage) who showed either right (above zero) or left (below zero) hand superiority in each subgroup (“left hand used”, “both hands used” and “non-musicians”) was also statistically tested (cross tabulation 2 × 3) for the T-FD (χ^2^_(Freeman−Halton)_ = 5.514, *p* = .056), the S-FD (χ^2^_(Freeman−Halton)_ = 1.754, *p* > .05), and the S-ED (χ^2^_(Freeman−Halton)_ = 15.284, *p* < .001), respectively. *P* was accepted as significant at 0.05/3 < 0.016, Bonferroni; (Fig. 3 – percentages in boxes). The above chi squared tests indicated that the majority of musicians who used primarily their left hand while playing were characterised by a left hand (non-dominant) superiority in the S-ED assessment. In contrast, the other two subgroups were characterised by the opposite effect.

A positive correlation between the T-FD asymmetry and the S-FD asymmetry was observed for non-musicians (*r* = .446, *p* < .05) but not for musicians (*r* = .125, *p* > .05). However, the correlation coefficients obtained from the two groups were not significantly different (Fisher r-to-z transformation, *z* = -1.15, *p* > .05). Finally, a negative correlation was also found between the S-ED asymmetry and age for non-musicians (*r*_*s*_ = − 0.472, *p* < .05) but not for musicians (*r*_*s*_ = − 0.232, *p* > .05), (Fisher r-to-z transformation, *z* = -2.42, *p* = .016, two-tailed). Data distribution was characterised by a slight curvilinearity; therefore, these correlations should be interpreted with caution. No significant correlations for musicians were found between any of the three asymmetry assessments and (a) the years of experience, (b) the age when started playing and (c) the cumulative hours of practicing.

## Discussion

The current study examined the hand asymmetry of the thickness and strength of the FD and the strength of the ED between musicians and non-musicians aiming to assess possible occupational effects on the muscular characteristics of musicians.

With respect to hand asymmetry, several studies so far have reported that the dominant hand has about 10% greater handgrip strength in right-handed participants. [25–27]. As expected, in the present study, in which all participants were right-handed, the asymmetry ratio for muscular strength and thickness in the non-musician group indicated larger values for the dominant hand with 4.3%, 7.5% and 6.3% for the T-FD, the S-FD and the S-ED respectively. Interestingly, in musicians, the asymmetry was less pronounced, 1.1%, 5% and − 0.2% for the same assessments respectively (Fig. 2). The clear tendency for musicians toward 0% indicates a more equal distribution between dominant and non-dominant hands. The tendency for more equally-distributed strength and muscle thickness became even more evident after categorizing musicians into those who primarily use fingers of their left hand (e.g., violinists) and those who use fingers of both hands to a similar degree (e.g., pianists). Musicians who typically use fingers of both hands to play showed the same pattern as non-musicians. In contrast, the majority of musicians who extensively use fingers of their left hand revealed a clear tendency towards less asymmetry or even a non-dominant hand superiority for the thickness flexor assessment (Fig. 3). The largest non-dominant hand superiority was observed for this group of musicians in the strength extension assessment (S-ED). The large effect of the strength extension toward non-dominant superiority can be attributed to the fact that playing a musical instrument requires considerable extension of the fingers. Compared to finger flexion movements, extension movements are less common during everyday activities, which could also explain why no differences were observed in the strength flexion assessment.

Similar results were found for the percentage of musicians who exhibited larger asymmetry toward the non-dominant hand; this effect was much larger for the “left hand used” group as compared to the other two groups (“both hands used” and “non-musicians”). This effect was obvious for the T-FD and significant for the S-ED (Fig. 3). The S-FD indicated no important differences concerning the percentage distribution across the three different groups. In particular, the proportion of hand superiority for non-musicians was relatively constant for all the assessments (~ 5:1, right: left hand superiority). These proportions are comparable to Incel et al. [27] who found that during a pulp pinch measurement of the first and second digit across 121 right-handed individuals, only 28% of participants were stronger in the non-dominant hand (4:1, right: left hand superiority). In contrast, for musicians who mostly use their left hand while playing, these proportions indicate superiority towards the left, non-dominant hand. For instance, the proportion for the S-ED was ~ 1:6, right: left hand superiority and for the T-FD it was ~ 1.3:1, right: left hand superiority. Our results suggest that right-handed musicians who play instruments requiring extensive usage of the left-hand fingers (e.g., violin) could have smaller (or even inverse) muscular thickness and strength extension asymmetries between the two hands. In contrast, musicians who extensively use fingers of both hands, such as pianists, exhibit muscular asymmetries more similar to non-musicians.

Different bilateral asymmetries due to the impact of the different instruments were also observed in skill activities and neural plasticity. For instance, Jäncke et al. [1] found that musicians exhibited reduced hand skill asymmetry compared to non-musicians. The authors argued that the reduced right-hand superiority was mainly an effect of extensive usage of the left hand and not due to a reduced skill of the right hand. Also, Bangert et al. [13] reported that the precentral gyrus in the right hemisphere of violinists was enlarged while it was smaller in keyboard players. The authors suggested that such structural brain differences might reflect an adaptation to the specific instrumental demands.

Finally, a positive correlation was observed in non-musicians between T-FD and S-FD asymmetry. A similar finding was also reported in the study of Abe and Loenneke [25] that examined a group of 31 young women and found that forearm muscle size was positively correlated with handgrip strength dominance. Notably, there was no sign for correlation in musicians and there was no significant difference between the two correlation coefficients. One explanation is that muscular mass is only partly or non-linearly related to muscular strength [28]. For instance, it has been suggested that a possible increase in strength could be achieved without any morphological changes such as muscle size. The increased muscular strength might be an adaptation of the motor system [29–31]. Past studies have already showed that strength training in childhood can increase muscular strength due to neuromuscular adaptations without hypertrophic factors [30, 32]. This finding could probably explain why extensive finger movements or increased finger strength can result in macroscopic adaptations in the nervous system of musicians [13] without any evidence of morphological changes in muscle thickness.

Muscular symmetry between the hands generally offers more advantages than disadvantages, particularly in the context of injury prevention and recovery from peripheral or central injuries. After an injury, symmetrical muscle development can facilitate recovery. If both hands (and associated musculature) are strong and well-coordinated, the unaffected hand can better assist and support the injured one during the rehabilitation process. For peripheral injuries (such as those affecting the limbs), balanced musculature can help distribute the workload during rehabilitation, allowing for more effective and less painful recovery. In the case of central injuries (such as those resulting from a stroke or spinal cord injury), muscular symmetry may aid in recovery as well. Symmetrical muscle development can support neuroplasticity, where the brain reorganizes itself by forming new neural connections [33]. Bilateral training can be particularly effective, helping the brain to recover motor functions by using both sides of the body in training [34]. Individuals with symmetrical muscle strength may find it easier to perform activities of daily living, as both hands are equally capable of performing tasks, leading to quicker and more comprehensive recovery of functional independence. However, individuals who engage in activities that demand high levels of bilateral muscle use might increase the risk of simultaneous or successive injuries to both sides. This is less common compared to asymmetric overuse injuries. Additionally, in the event of an injury, individuals with symmetrical muscle development might unintentionally overcompensate with the uninjured side, potentially leading to overuse injuries if not managed properly. In general, muscular symmetry supports balanced strength and coordination, which can contribute to more efficient and effective motor function, beneficial in daily activities and sports. Symmetrical muscular development can reduce the risk of overuse injuries, as there is less strain on individual muscles and joints when one side of the body is not compensating for the other [35].

Future studies could recruit a larger number of musicians to examine further occupational differences not only from hand but also from facial muscles, for instance in wind players. In addition, a sample derived from a broader age range of musicians can provide us with information on when muscular asymmetry first becomes apparent, how it sustains over the years, and how it behaves in older ages where the hours of practice gradually decrease. Finally, an interesting approach would be to investigate differences between musicians and people from other occupations requiring extensive use of the fingers, such as typists. This will enhance our understanding on how small muscles either in the forearm or around the mouth adapt to the different occupational demands.

## Conclusions

Our study revealed that musicians, in contrast to non-musicians, demonstrate reduced hand-by-hand asymmetry in both FD thickness and strength, as well as in ED strength. This suggests that the FD may adjust to the demands of playing various musical instruments, with certain instrumentalists displaying heightened superiority in their non-dominant hand. This phenomenon is likely attributed to the prolonged engagement with musical instruments. These findings imply that, before conducting clinical examinations or studies involving muscular asymmetries, it is crucial to consider the individuals’ occupations to establish accurate baseline measurements.

### Electronic supplementary material

Below is the link to the electronic supplementary material.

Supplementary Material 1

## Data Availability

Data availble uppon request.

## Abbreviations

ED: Extensor digitorum
T-FD-1: First thickness assessment of the flexor digitorum
FD: Flexor digitorum
T-FD-2: Second thickness assessment of the flexor digitorum
S-FD: Strength of the flexor digitorum
T-FD: Thickness of the FD
