# Supplementary material for: The impact of fine motor activities like playing musical instruments on the thickness and strength of the flexor digitorum muscle
Source: J Occup Med Toxicol. 2024 Aug 14;19:34. doi: 10.1186/s12995-024-00430-9 (PMC11323442; doi:10.1186/s12995-024-00430-9)
Supplement: Supplementary file 1 — Supplementary Material 1 [file 12995_2024_430_MOESM1_ESM.docx]

# APPENDICES


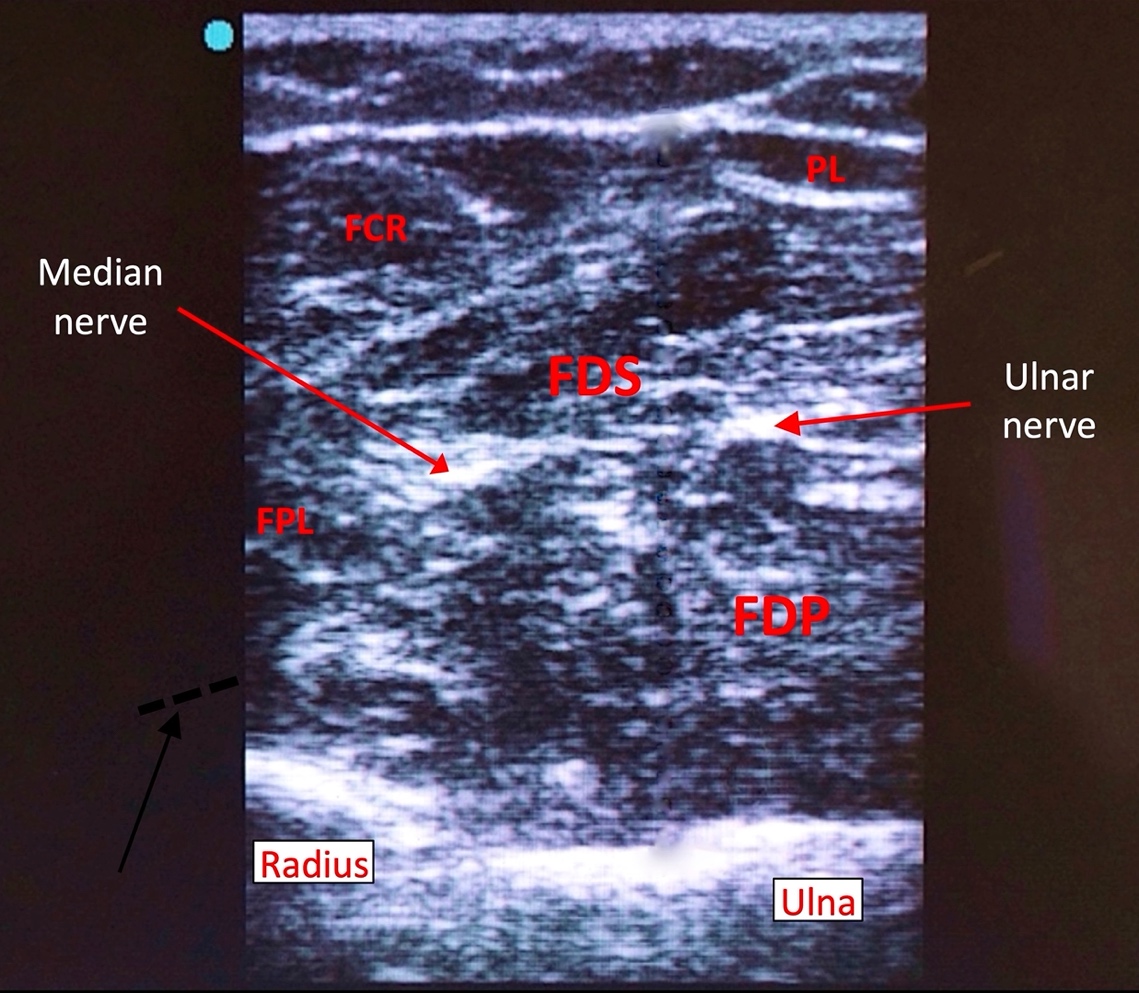


Figure A. This is an ultrasound image example indicating that the borders between the superficial and profound part of the flexor digitorum cannot be clearly identified via ultrasonography. Abbreviations: FDS = flexor digitorum superficial; FDP = flexor digitorum profound; FCR = flexor carpi radialis; FPL = flexor pollicis longus; PL = palmaris longus.
